# UVB protective effects of *Sargassum horneri* through the regulation of Nrf2 mediated antioxidant mechanism

**DOI:** 10.1038/s41598-021-88949-3

**Published:** 2021-05-11

**Authors:** Eui Jeong Han, Seo-Young Kim, Hee-Jin Han, Hyun-Soo Kim, Kil-Nam Kim, Ilekuttige Priyan Shanura Fernando, Disanayake Mudiyanselage Dinesh Madusanka, Mawalle Kankanamge Hasitha Madhawa Dias, Sun Hee Cheong, Sang Rul Park, Young Seok Han, Kyounghoon Lee, Ginnae Ahn

**Affiliations:** 1 Research Center for Healthcare and Biomedical Engineering, Chonnam National University, Yeosu, 59626 Republic of Korea; 2 Department of Food Technology and Nutrition, Chonnam National University, Yeosu, 59626 Republic of Korea; 3 Chuncheon Center, Korea Basic Science Institute, Chuncheon, 24341 Republic of Korea; 4 National Marine Biodiversity Institute of Korea, Janghang-eup, Seocheon, 33662 Republic of Korea; 5 Control Center for Aquatic Animal Diseases, Chonnam National University, Yeosu, 59626 Republic of Korea; 6 Department of Marine Bio-Food Sciences, Chonnam National University, Yeosu, 59626 Republic of Korea; 7 Estuarine and Coastal Ecology Laboratory, Department of Marine Life Sciences, Jeju National University, Jeju, 63243 Republic of Korea; 8 Neo Environmental Business Co., Daewoo Technopark, Doyak-ro, Bucheon, 14523 Republic of Korea; 9 Division of Fisheries Science, Chonnam National University, Yeosu, 59626 Republic of Korea; 10 Department of Marine Technology, Chonnam National University, Yeosu, 59626 Republic of Korea

**Keywords:** Cell death, Cell signalling, Biochemistry, Cell biology

## Abstract

The present study aimed to evaluate the protective effect of a methanol extract of *Sargassum horneri* (SHM), which contains 6-hydroxy-4,4,7a-trimethyl-5,6,7,7a-tetrahydrobenzofuran-2(4H)-one (HTT) and apo-9′-fucoxanthinone, against ultraviolet B (UVB)-induced cellular damage in human keratinocytes and its underlying mechanism. SHM significantly improved cell viability of UVB-exposed human keratinocytes by reducing the generation of intracellular reactive oxygen species (ROS). Moreover, SHM inhibited UVB exposure-induced apoptosis by reducing the formation of apoptotic bodies and the populations of the sub-G_1_ hypodiploid cells and the early apoptotic cells by modulating the expression of the anti- and pro-apoptotic molecules, Bcl-2 and Bax, respectively. Furthermore, SHM inhibited NF-κB p65 activation by inducing the activation of Nrf2/HO-1 signaling. The cytoprotective and antiapoptotic activities of SHM are abolished by the inhibition of HO-1 signaling. In further study, SHM restored the skin dryness and skin barrier disruption in UVB-exposed human keratinocytes. Based to these results, our study suggests that SHM protects the cells against UVB-induced cellular damages through the Nrf2/HO-1/NF-κB p65 signaling pathway and may be potentially useful for the prevention of UVB-induced skin damage.

## Introduction

Mammalian skin is an essential organ of the integumentary system, which guards the underlying muscles, bones, ligaments, and internal organs. Being the interface between body and environment, the skin is the first line of defense against various external stimuli such as pathogens and ultraviolet (UV)-radiation, and acts as a barrier preventing excessive water loss. Frequent exposure to UV radiation results in photo-aging of the skin^1^. Based on their wavelength, UV radiations are classified into three types, UVA (320–400 nm), UVB (290–320 nm), and UVC (100–290 nm). Generally, UVB exposure, comprising 5–10% of all UV wavelengths penetrate the basal cell layer of the epidermal cells including keratinocytes, especially stratum corneum and triggers responses leading to photoaging of the skin by inducing the abnormal ROS generation^2–5^. UVB-induced oxidative stress leads to mitochondria-mediated apoptosis, regulated by key mediators, including Bcl-2 and Bax^6^. UVB frequently causes mutations in the p53 gene, which is a faulty trigger of apoptosis in keratinocytes and promotes non-melanoma skin cancers^7^. The previous study also reported that DNA damage induces apoptosis through the tumor suppressor protein, p53, which in turn causes cell cycle arrest, while triggering cellular repair systems^8^. Moreover, recent studies have indicated that UVB exposure can cause the dysfunction of skin epidermal barrier and skin dryness and finally skin aging and cancers^4,9^.

Many studies have shown that the nuclear factor erythroid-derived 2-related factor 2 (Nrf2)/heme oxygenase-1 (HO-1) signaling plays a vital role in response to oxidative stress and cytoprotection^10^. Under healthy or non-stressed conditions, Nrf2 is retained in the cytoplasm in association with its negative regulator Kelch-like epichlorohydrin-associated protein 1 (Keap1) and Cullin 3 (Cul3)^10,11^. However, oxidative stress modifies the cysteine residues in Keap1 and disrupts the Keap1-Cul3 ubiquitination system leading to translocation of Nrf2 from the cytoplasm into the nucleus^11,12^. In the nucleus, Nrf2 binds to the antioxidant response element (ARE) in the upstream promoter region of antioxidant genes and initiates their transcription^10,13^. HO-1 is a critical antioxidant enzyme that regulates intracellular ROS levels in response to diverse stimuli. Its transcription is regulated by Nrf2^14^. In addition to their role as antioxidant enzymes, the Nrf2/HO-1 pathway proteins also exhibit anti-inflammatory functions through nuclear factor kappa-light-chain-enhancer of activated B cells (NF-κB) signaling^14^. Under non-stress conditions, NF-κB proteins reside in the cytoplasm, associated with the inhibitory factor, inhibitor of nuclear factor kappa B (IκB)^14^. NF-κB is activated following phosphorylation and proteolytic degradation of IκB^14^. Activated NF-κB is translocated into the nucleus, subsequently activating immune response genes^15^.

*Sargassum horneri* (*S*. *horneri*) is an edible brown seaweed abundant in the Asia-Pacific region^16^. Secondary metabolites of *S*. *horneri* are reported to possess numerous biological activities^16–19^. In particular, *S*. *horneri* and its active componants such as polysaccharides, 6-hydroxy-4,4,7a-trimethyl-5,6,7,7a-tetrahydrobenzofuran-2(4H)-one (HTT) and apo-9′-fucoxanthinone exhibit antioxidant and anti-inflammatory activities^20–23^. However, there have been no reports on protective effect of *S. horneri* in UVB-exposed keratinocytes and its biological mechanism.

Therefore, we investigated whether a methanol extract of *S*. *horneri* (SHM) has a protective effect against UVB-induced cellular damages in human keratinocytes and its biological mechanism.

## Materials and methods

### Materials

HaCaT cells, human keratinocytes were kindly supplied by Surface Science Laboratory of Center for Anti-Aging Molecular Science of the Department of Chemistry and School of Molecular Science of Korea Advanced Institute of Science and Technology^24^. Dulbecco’s modified Eagle’s medium (DMEM), fetal bovine serum (FBS), and penicillin/streptomycin were purchased from Thermo Fisher Scientific Inc. (Waltham, MA, USA). 2′,7′-dichlorodihydroflurescin diacetate (DCFH-DA), 3-(4-5-dimethyl-2yl)-2-5-diphynyltetrasolium bromide (MTT), dimethyl sulfoxide (DMSO), Hoechst 33342, and propidium iodide (PI) were purchased from Sigma-Aldrich (St, Louis, MO, USA). Primary and secondary antibodies were purchased from Cell Signaling Technology Inc. (Danvers, MA, USA), and β-actin was purchased from Sigma-Aldrich. Other chemicals and reagents were of the highest grade available commercially.

### Preparation of methanol extract from *S*. *horneri* (SHM)

*S. horneri* was obtained from Jeju, South Korea. Samples were washed to remove impurities, freeze-dried, powdered, and extracted (50 g) using 1 L of 80% methanol by continuous agitation for 24 h at room temperature (25 °C) according to the method described by Kim et al.^21^. The extraction was performed thrice. Finally, the extract was filtered and concentrated using a rotary evaporator to obtain a crude extract, SHM.

### Carbohydrate, protein, total phenolic, and flavonoid contents in SHM

The carbohydrate, protein, total phenolic, and flavonoid contents in SHM were measured according to the method described in a previous study^25,26^.

### Cytoprotective effect of SHM on UVB-exposed human keratinocytes

To verify the cytoprotective effect of SHM on UVB-exposed human keratinocytes, the viability of cells was evaluated using MTT assay. Human keratinocytes (1 × 10^5^ cells/well) were treated with various concentrations of SHM (31.6, 62.5, and 125 µg/mL) at 37 °C for 2 h and exposed to UVB (40 mJ/cm^2^). After a 48 h incubation period, the cells were treated with MTT stock solution (5 mg/mL) for 4 h and the resulting formazan crystals were dissolved in DMSO. The absorbance was measured at 570 nm using a microplate reader (SpectraMax M2/M2e, CA, USA). Cell viability was calculated using the following formula:

$${\text{Cell viability }}\left( \% \right) \, = {\text{ B or C}}/{\text{A}}*{1}00$$

A: absorbance of control cells; B: absorbance of only UVB-exposed cells; C: absorbance of UVB-exposed and sample-treated cell.

### Effect of SHM on intracellular ROS generation in UVB-exposed human keratinocytes

To examine the effect of SHM on the generation of intracellular ROS in UVB-exposed human keratinocytes, an oxidation-sensitive fluorescent dye, 2′,7′-dichlorofluorescein diacetate (DCFH-DA) was used. Following cell uptake, DCFH-DA is deacetylated by nonspecific cellular esterase to a non-fluorescent compound, which is further oxidized by ROS into the highly fluorescent compound, dichlorofluorescein (DCF)^27^. Human keratinocytes (1 × 10^5^ cells/well) were pretreated with SHM (31.6, 62.5, and 125 µg/mL) for 2 h and exposed to UVB for an additional 1 h at 37 °C. The generated intracellular ROS were detected using DCFH-DA at the excitation and emission spectra of 485 nm and 520 nm, respectively, using a microplate reader (SpectraMax M2/M2e).

### Effect of SHM on apoptotic body formation in UVB-exposed human keratinocytes

Hoechst 33342 staining was performed to determine whether SHM reduced the UVB-induced formation of apoptotic bodies in human keratinocytes. Human keratinocytes (3 × 10^5^ cells/well) were pretreated with SHM (31.6, 62.5, and 125 µg/mL) for 2 h and exposed to UVB (40 mJ/cm^2^) at 37 °C. After 24 h, the cells were stained with Hoechst 33342 reagent (2 µg/mL) at 37 °C for 30 min. The nuclear morphology of the stained cells was examined using a fluorescence microscope (Olympus, Shinjuku, Japan).

### Effect of SHM on the accumulation of sub-G_1_ hypodiploid cells in UVB-exposed human keratinocytes

Flow cytometry was used to evaluate the effect of SHM on sub-G_1_ DNA content in UVB-exposed human keratinocytes. Human keratinocytes (3 × 10^5^ cells/well) were pretreated with SHM (31.6, 62.5, and 125 µg/mL) for 2 h and exposed to UVB at 37 °C. After 24 h, the cells were stained with PI (500 µL containing 50 µg/mL in 2 mM PBS-EDTA and RNase A [0.2 µg/mL; Promega, WI, USA]). The sub-G_1_ DNA content of the cells was measured using a CytoFLEX (Beckman coulter, Brea, CA, USA).

### Effect of SHM on the expression of apoptotic mediators, the activation of Nrf2/HO-1/NF-κB signaling, skin moisture and skin barrier factors in UVB-exposed human keratinocytes

Western blot analysis was performed to investigate the effect of SHM on the expression of apoptotic mediators, Nrf2/HO-1, and NF-κB signaling. Human keratinocytes (1 × 10^6^ cells/well) were pretreated with SHM (31.6, 62.5, and 125 µg/mL) for 2 h and exposed to UVB for 24 h (apoptosis mediators, and skin moisture and skin barrier), 30 min (Nrf2/HO-1 signaling) and 1 h (NF-κB) at 37 °C. Cytoplasmic and nucleic proteins were extracted from the cells using a nuclear and cytoplasmic extraction kit (Thermo Fisher Scientific Inc.), and the proteins (40 μg) were subjected to western blot analysis. The primary antibodies used in this study were anti-Bcl-2, Bax, p53, Nrf2, HO-1, IκBα, p-IκBα, NF-κB p65, p-NF-κB p65, LEKT1, KLK5, PLA-2, PAR-2, involucrin, filaggrin, occludin, ZO-1, and claudin 1, 4, 7 and 23 antibodies (all used at 1:1000 dilution, Cell Signaling Technology Inc.). Primary anti-β-actin antibody (1:3000 dilution) was from Sigma-Aldrich. The secondary antibodies were HRP-conjugated anti-mouse IgG and anti-rabbit IgG antibodies (1:5000, Cell Signaling Technology Inc.). The protein bands were detected using an enhanced SuperSignal West Femto Maximum Sensitivity Substrate (Thermo Fisher Scientific Inc.) and densitometric analysis of western blot bands were carried out using NIH Image J software 1.52a (National Institute of Health, Bethesda, MD, USA)^28^.

### Influence of HO-1 inhibition on the UVB protective activity of SHM in human keratinocytes

To investigate whether HO-1 activation was required for UVB protective effect of SHM in human keratinocytes, MTT assay, DCFH-DA assay, Annexin V/PI staining and western blot analysis were performed. Human keratinocytes were pretreated with 5 μM Zinc protoporphyrin (ZnPP; a HO-1 inhibitor) for 2 h with or without SHM and exposed to UVB. MTT assay, DCFH-DA assay, and western blot analysis were performed by the methods described in the above section. To evaluate effect of SHM on apoptotic cell population with/without ZnPP in UVB-exposed human keratinocytes, we used an Annexin V-conjugated fluorescein isothiocyanate and propidium iodide kit (Invitrogen, CAT #V13242, Carlsbad, CA, USA) following the manufacturer's instructions.

### Statistical analysis

Data were analyzed using the SPSS package (Version 21). Values are expressed as mean ± standard error (SE). The mean values of the tail intensity from each treatment were compared using one-way analysis of variance (ANOVA) followed by Duncan’s multiple range test. A *p*-value less than 0.01 was considered significant.

## Results

### Chemical composition of SHM

We first analyzed the chemical composition of SHM. As shown in Table 1, SHM contained the following contents: total phenolic compounds (7.45 ± 0.29%), protein (4.86 ± 0.14%), carbohydrate (1.49 ± 0.01%), and flavonoid compounds (0.29 ± 0.07%).

**Table 1:** Yield and composition of major chemical constituents in the methanol extract of *S. horneri*.

Sample	Yield	Composition of contents (%)			
		Protein	Carbohydrate	Phenol	Flavonoids
SHM	7.78 ± 0.60	4.86 ± 0.14	1.49 ± 0.01	7.45 ± 0.29	0.29 ± 0.07

### Effect of SHM on cell viability and ROS production in UVB-exposed human keratinocytes

**Figure 1:**
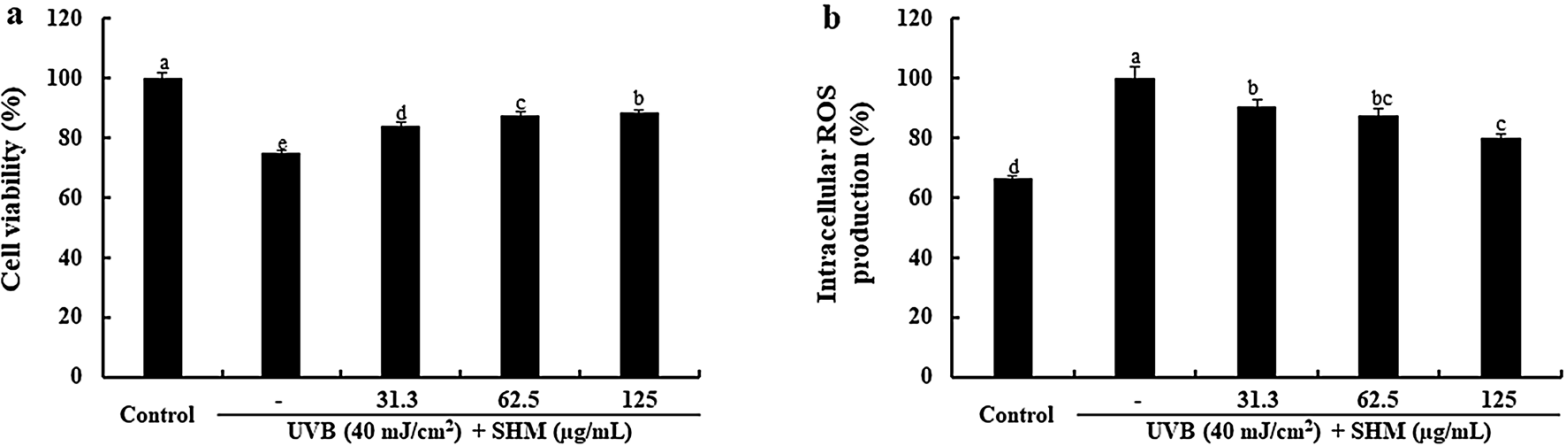
Cytoprotective effect of SHM on cell viability (**a**) and intracellular ROS production (**b**) in UVB-exposed human keratinocytes. Values are expressed as means ± SE of triplicate determinants (n = 3). ^a–e^The bars with different letters represent significant differences (p < 0.01).

To determine the appropriate concentration of SHM for evaluation of its protective effect on UVB-exposed human keratinocytes (the main cell types in the basal layer of the skin), we evaluated its cytotoxic effect using the MTT assay. The results showed that SHM did not have any cytotoxic effect at the concentrations tested (31.6, 62.5, and 125 µg/mL) in human keratinocytes (data not shown). Therefore, we used all three concentrations of SHM for further studies. We first sought to determine the effect of SHM on cell viability following UVB exposure. As shown in Fig. 1a, UVB exposure markedly reduced the viability of human keratinocytes up to 74.57 ± 1.05%, compared to that of the untreated and non-exposed cells. Interestingly, pretreatment with SHM improved the cell viability, which was reduced following UVB exposure, in a concentration-dependent manner. In addition, as shown in Fig. 1b, the intracellular ROS generation levels were increased by 40% in the UVB-exposed cells compared to the non-exposed cells, whereas they were significantly reduced following the pretreatment with SHM. These results demonstrate that SHM improves the cell viability with an ROS scavenging effect following UVB exposure in human keratinocytes.

### Protective effect of SHM against UVB exposure-mediated apoptosis

**Figure 2:**
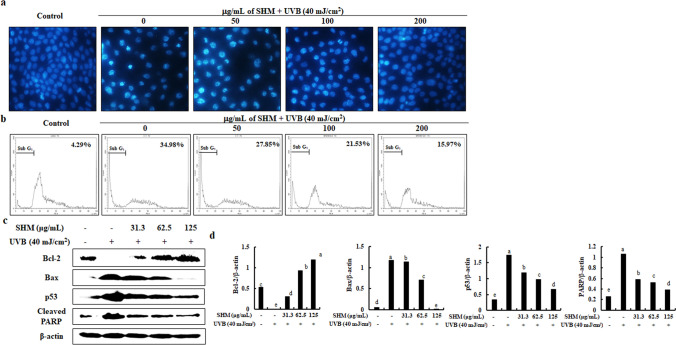
Effect of SHM on apoptosis signaling in UVB-exposed human keratinocytes. (**a**) Apoptotic body formation in SHM-pretreated and UVB-exposed human keratinocytes. The cells were stained with Hoechst 33342 reagent (2 µg/mL) and examined using a fluorescence microscope. (**b**) Analysis of Sub-G_1_ apoptotic populations in SHM-pretreated and UVB-exposed human keratinocytes by flow cytometry using propidium iodide (PI). (**c**) The expression levels of mitochondria-mediated apoptotic molecules (Bcl-2, Bax, p53, and PARP) in SHM-pretreated and UVB-exposed human keratinocytes. (**d**) The density ratio of protein expression levels was analyzed via Image J software each in comparison to β-actin. Values are expressed as means ± SE of triplicate determinants (n = 3). ^a–e^Bars with different letters for each molecule represent significant differences (P < 0.01).

To analyze the protective effect of SHM on UVB-mediated cell damages, the nuclei of human keratinocytes were stained with Hoechst 33342 to identify the formation of apoptotic bodies. As shown in Fig. 2a, photomicrographs showed non-treated cells with intact nuclei, whereas the UVB-exposed cells exhibited significant fragmentation and nuclear condensation, characteristic of apoptosis. When the cells were treated with SHM, the amount of fragmentation and nuclear condensation in the UVB-exposed cells was markedly reduced. In addition, we investigated the effects of SHM on the increment of sub-G_1_ population caused by UVB exposure. Figure 2b showed that (4.29%), UVB exposure cause the increment of the sub-G_1_ population (34.98%), indicative of apoptotic cells in human keratinocytes, compared to the non-exposed control cells. However, pretreatment with SHM (31.6, 62.5, and 125 µg/mL) reduced the population of sub-G_1_ phase from 27.85 to 15.97% in a dose-dependent manner. Collectively, these results indicate that SHM exhibits the anti-apoptotic activity via the reduction of apoptosis in UVB-exposed human keratinocytes.

### SHM reduces UVB exposure-induced apoptosis through the regulating mitochondria-mediated apoptotic pathway

To confirm the effect of SHM on the expression of the anti-apoptotic protein, Bcl-2, and pro-apoptotic proteins, Bax, p53, and PARP in UVB-exposed human keratinocytes, we performed the western blot analysis. As shown in Fig. 2c,d, SHM pretreatment markedly increased the expression of Bcl-2 protein, which was reduced following UVB exposure. In contrast, the expression levels of the pro-apoptotic proteins (Bax, p53, and PARP), which increased following UVB exposure, were significantly downregulated by pretreatment with SHM. These results suggest that SHM inhibits apoptosis by regulating the mitochondrial apoptotic pathway activated by UVB exposure in human keratinocytes.

### SHM inhibits the activation of NF-κB signaling in UVB-exposed human keratinocytes

**Figure 3:**
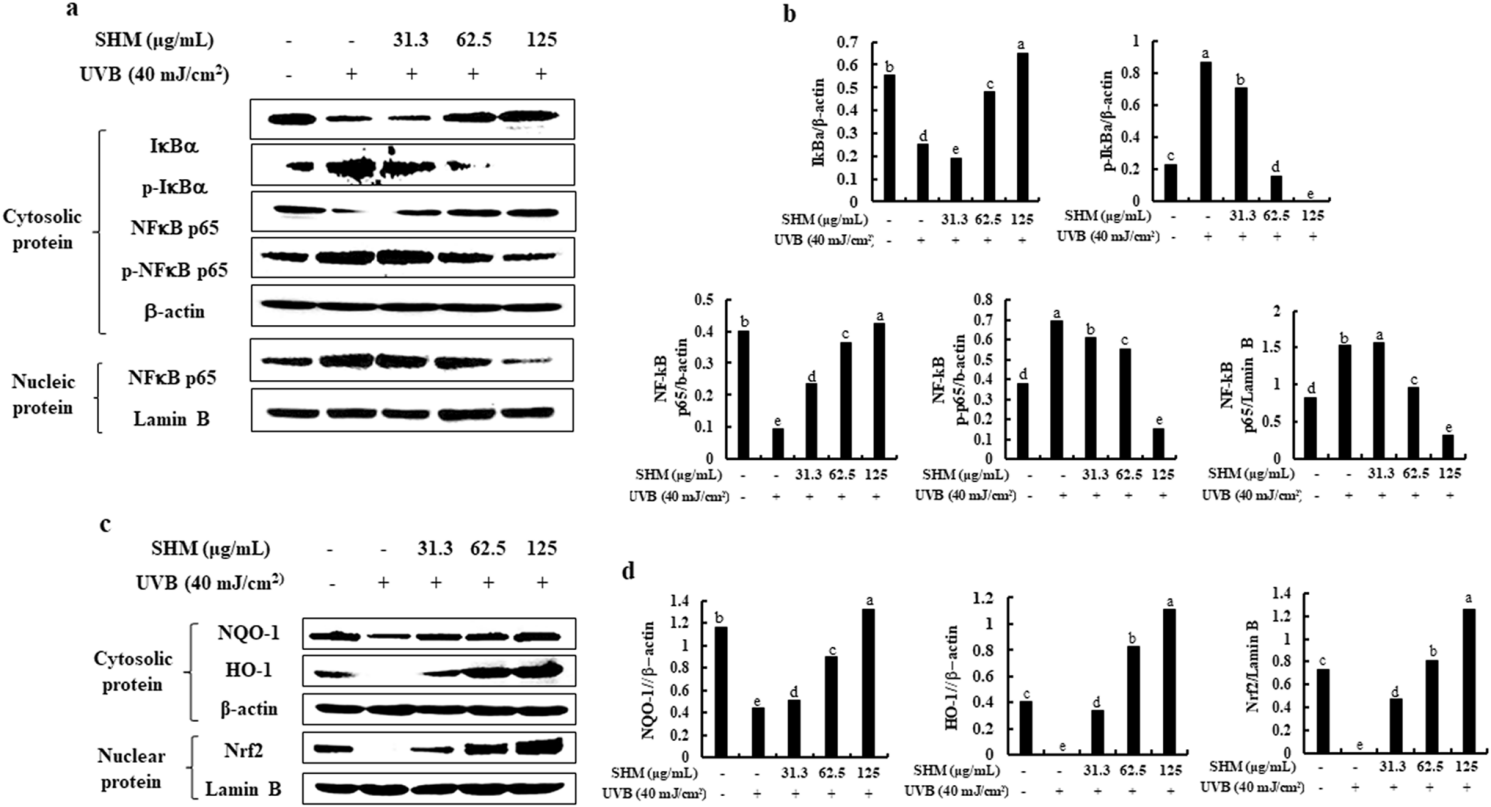
Effect of SHM on the activation of NF-κB and the reduction of Nrf2/HO-1 in UVB-exposed human keratinocytes. (**a**) NF-κB-related protein expression levels in SHM-pretreated and UVB-exposed human keratinocytes. (**c**) Nrf2/HO-1-related protein expression levels in SHM-pretreated and UVB-exposed human keratinocytes. (**b**,**d**) The density ratio of protein expression was analyzed via Image J software each in comparison to β-actin or Lamin B. Values are expressed as means ± SE of triplicate determinants (n = 3). ^a–e^Bars with different letters for each molecule are significantly different (P < 0.01).

To determine whether SHM inhibits UVB exposure-induced activation of NF-κB signaling, we performed western blot analysis. Figure 3a,b reported that UVB exposure markedly induced the degradation and phosphorylation of IκBα and NF-κB p65 in the cytosol as well as the translocation of NF-κB p65 into the nucleus, compared to the non-exposed and non-treated control cells. Interestingly, these effects were dose-dependently abolished by pretreatment of SHM. Based on these results, we indicate that SHM effectively suppresses the activation of NF-κB signaling in UVB-exposed human keratinocytes.

### Inhibition of HO-1 abolishes the cytoprotective and antiapoptotic effects of SHM in UVB-exposed human keratinocytes

**Figure 4:**
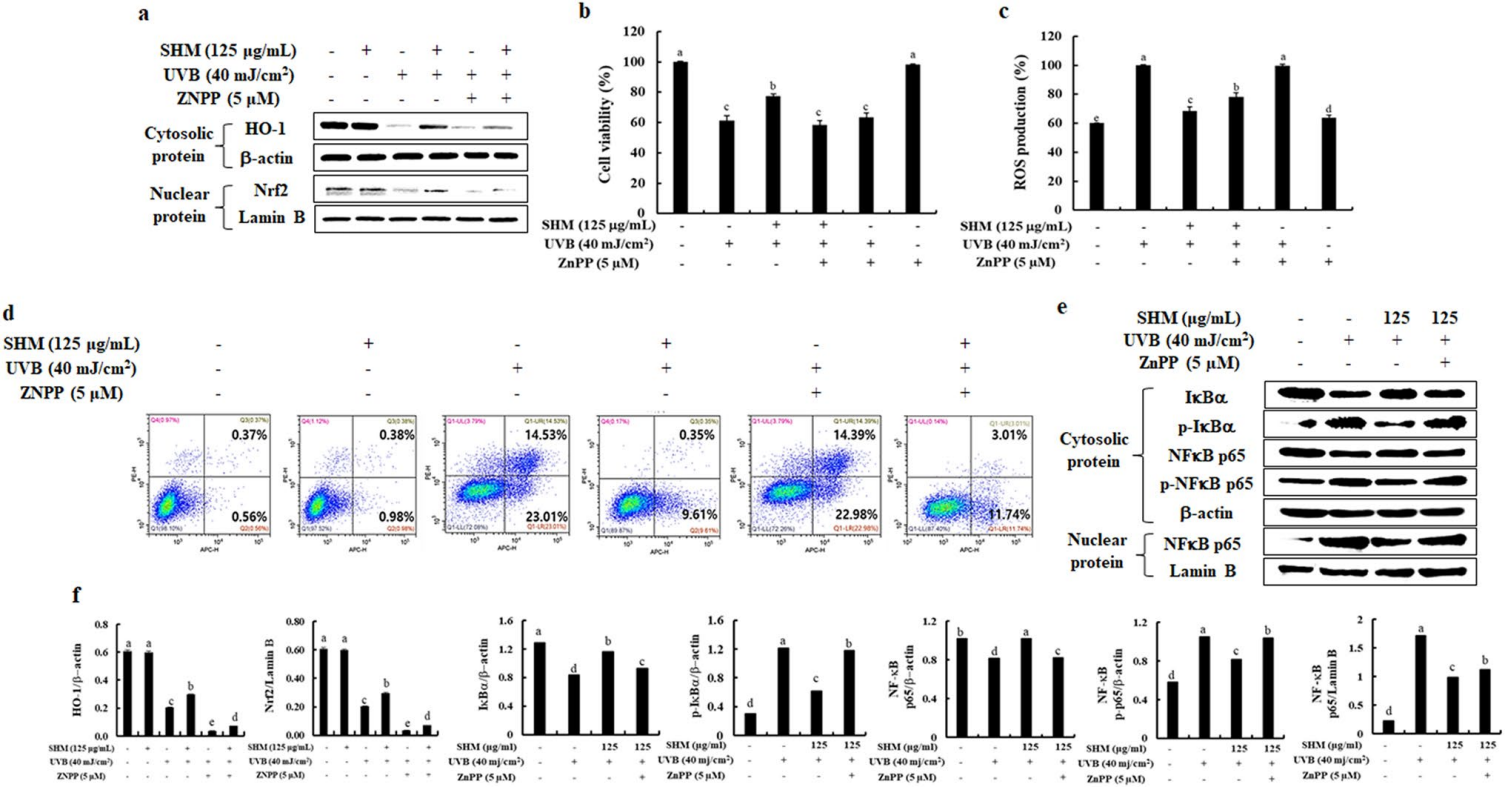
Influence of HO-1 inhibition on the protective effects of SHM in UVB-exposed human keratinocytes. Influence of HO-1 inhibition on (**a**) Nrf2/HO-1-related protein expression level, (**b**) cell viability, (**c**) intracellular ROS production, (**d**) population of early apoptotic and necrotic cells, (**e**) NF-κB-related protein expression levels in SHM-pretreated and UVB-exposed human keratinocytes, and (**f**) The density ratio of protein expression levels was analyzed via Image J software each in comparison to β-actin or Lamin B. Values are expressed as means ± SE of triplicate determinants (n = 3). ^a–e^Bars with different letters for each molecule represent significant differences (P < 0.01).

Next, we evaluated the effect of ZnPP, a HO-1 inhibitor in blocking SHM’s action on the cytoprotective and antiapoptotic effects in UVB-exposed human keratinocytes. First of all, we discovered SHM induced the activation of HO-1/Nrf2 reduced by UVB exposure in human keratinocytes, whereas the SHM-induced HO-1 activation was inhibited by the HO-1 inhibition in UVB-exposed human keratinocytes (Figs. 3c,d, 4a,f). As shown in Fig. 4b,c, SHM effectively increased the decreased cell viability in UVB-exposed human keratinocytes, whereas reduced the increased intracellular ROS generation. Interestingly, when we added ZnPP, a HO-1 inhibitor into the SHM-treated and UVB-exposed cells, the capacities of SHM on the reduction of intracellular ROS generation and the increment of cell viability were partially abolished, although it was not perfect. Next, we performed the Annexin V/PI staining to evaluate the contribution of Nrf2-HO-1 activation for the anti-apoptotic activity of SHM. Normally, fluorochrome conjugated Annexin V can be used to detect early apoptotic cells by flow cytometry. As shown in the below figure (Fig. 4d), UVB exposure increased the populations of early apoptotic cells (23.01 ± 0.12%) and necrotic cells (14.53 ± 0.15%), compared to non-treated cells (0.56 ± 0.24% and 0.37 ± 0.17%, respectively), whereas they were effectively reduced by the pretreatment of SHM to 9.61 ± 0.17% and 0.35 ± 0.20%, respectively. Interestingly, the SHM’s anti-apoptotic abilities were partially abolished by the blockage of HO-1 in UVB-exposed human keratinocytes (11.74 ± 0.18% of early apoptotic cell population and 3.01 ± 0.11% of necrotic cell population, respectively). Moreover, the additional results exhibited the application of ZnPP, an inhibitor of HO-1, blocked the NF-kB activation inhibited by SHM in UVB-exposed human keratinocytes as indicated in Fig. 4e,f. From these results, we suggest that the activation of HO-1/Nrf2 is required for the cytoprotective and antiapoptotic effects of SHM in UVB-exposed human keratinocytes.

### SHM modulated the expression levels of skin moisture and skin barrier factors in UVB-exposed human keratinocytes

**Figure 5:**
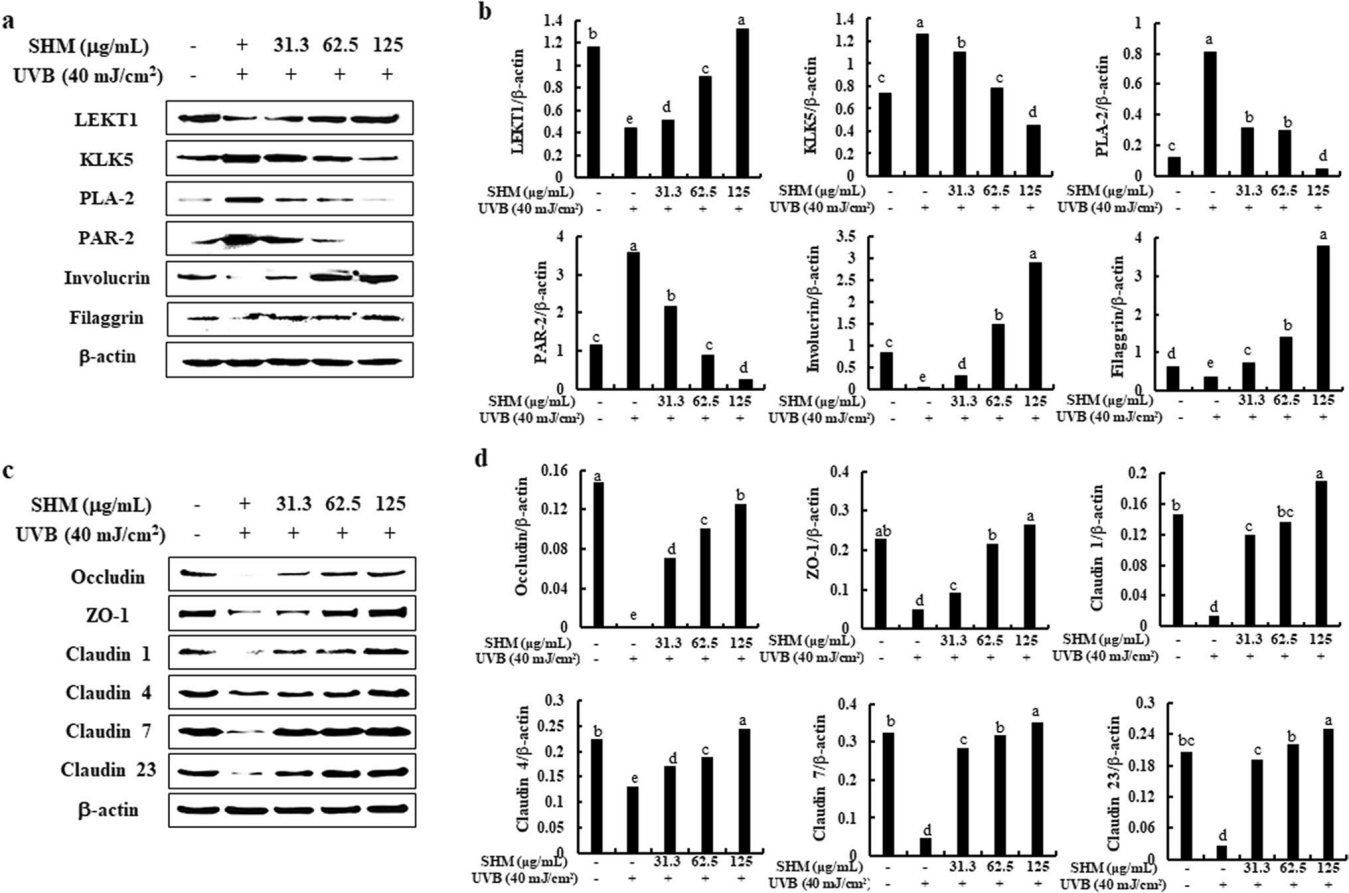
Effects of SHM on UVB exposure-induced skin dryness and skin barrier disruption. (**a**) Skin moisturization-related protein expression levels, (**c**) skin barrier-related protein expression levels, (**b**,**d**) the density ratio of protein expression was analyzed via Image J software each in comparison to β-actin. All experiments were performed in triplicate. ^a–e^Bars with different letters for each molecule represent significant differences (P < 0.01).

We investigated whether SHM affect to the skin moisture and skin barrier factors in UVB-exposed human keratinocytes. Figure 5a exhibited that UVB exposure significantly decreased the expression levels of LEKT1, involucrin and filaggrin, which are essential for the formation of the epidermal barrier, while increased those of KLK5, PLA-2 and PAR-2, which cellular adherence, and regulate skin shedding^29^. Interestingly, they were effectively modulated by the pre-treatment of SHM (Fig. 5a,b). In addition, SHM increased the protein expression levels of skin barrier molecules including occludin, ZO-1, claudin 1, claudin 4, claudin 7 and claudin 23 decreased by UVB exposure (Fig. 5c,d). These results demonstrated that SHM restored the skin dryness skin barrier disruption in UVB-exposed human keratinocytes.

## Discussion

The present study first indicates the protective effect of *S*. *horneri* in UVB-induced cellular damages in human keratinocytes and its biological mechanism. Here, we revealed the methanol extract of *S. horneri*, SHM had the cyto-protective and the anti-apoptotic effects against cellar damages and enhanced skin barrier function and skin moisture in UVB-exposed human keratinocytes.

Skin lesions caused by UVB exposure involves the generation of free radicals such as singlet oxygen, hydrogen peroxide, superoxide anion, and hydroxyl radicals while altering antioxidant defensive systems^27^. UVB-induced free radicals lead to the accumulation of excessive ROS that are toxic to skin keratinocytes and dermal fibroblast cells^18^. These ROS cause oxidative stress and cellular damage resulting in apoptosis and reduced cell viability^30^. Excessive ROS levels also exert adverse effects on cells by initiating cell death pathways. In particular, the reduced viability and increased ROS generation in keratinocytes play an important role in the destruction and damage to the skin^31^. Interestingly, our report also showed that the pretreatment of SHM increased the cell viability by reducing the intracellular ROS generation in UVB-exposed human keratinocytes. Generally, Nrf2-mediated HO-1 signaling pathway is an essential regulator of cellular oxidative stress^10^. Once stimulated, the Nrf2 is existed in the cytosol translocate into the nucleus, where it binds to the ARE and initiates the expression of cytoprotective enzymes and antioxidants such as HO-1^32^. HO-1 metabolizes heme to generate carbon monoxide (CO), biliverdin, and iron. HO-1 level is a critical measure of the anti-cancer, anti-inflammatory, anti-apoptotic, antiproliferative, and antioxidant effects of a drug^33^. As indicated in Fig. 3c,d, SHM induced the activation of Nrf2-mediated HO-1 signaling pathway via the increment of cytoplasmic HO-1 expression and the translocation of Nrf2 into the nucleus in UVB-exposed human keratinocytes. These results demonstrate that the antioxidant effect of SHM including the ROS scavenging capacity contributed to the improvement of the cell viability and led to the cyto-protective effects in UVB-exposed human keratinocytes.

DNA damage caused by UVB-exposure is one of the most sensitive biological markers for evaluating oxidative stress, which represents an imbalance between ROS generation and the efficacy of the antioxidant system^27^. Hoechst 33342 staining and PI staining were typical analysis for identifying the DNA damages in cells^27^. Hoechst 33342 staining was an assay that could visually confirm the apoptosis of cells and following Hoechst 33342 staining, cells with homogeneously stained nuclei are considered viable, whereas the presence of chromatin condensation and/or fragmentation is indicative of apoptosis^34^. In addition, cell cycle arrest through PI staining is considered the major anti-proliferation marker^35^. Flow cytometry allows for rapid cell cycle analysis. Cells that have lost intact DNA take up less stain and appear to the left of the G_1_ peak; hence, the size of the G_1_ peak in the histogram is directly proportional to the apoptosis induced by a given treatment^36^. Annexin V/PI dual staining is one of the gold standard technique to detect early apoptotic cells^37^. We also identified that SHM inhibited UVB exposure caused apoptosis such as the formation of apoptotic body, increment of the early apoptotic and necrotic cell population in human keratinocytes. Thus, our results suggested that SHM effectively inhibited UVB exposure caused cell apoptosis such as the formation of apoptotic body and the increment of the early apoptotic and necrotic cell population in human keratinocytes. In the mitochondrial apoptotic pathway, the two typical members of the Bcl family of proteins, Bcl-2 and Bax, play an essential role in inhibiting or promoting apoptosis^6^. Bcl-2 is an upstream molecule in the apoptotic pathway and a potent suppressor of apoptosis^36^ whereas Bax is essential for propagating death receptor-mediated apoptosis^30^. Following DNA damage, p53 arrests cells in the G_1_ stage until the DNA is repaired. When the DNA damage is too severe or the DNA repair fails, then p53 acts as an inducer of apoptosis^7^. Cleavage of PARP facilitates cellular disassembly and serves as a marker of cells undergoing apoptosis^33^. Figure 2c showed that SHM up-regulated the protein expression level of anti-apoptotic molecule, Bcl-2, whereas down-regulated those of pro-apoptotic molecules, Bax, p53 and cleaved PARP in UVB-exposed human keratinocytes. With these results, we revealed that SHM protects the cells by regulating apoptosis via the inhibition of the mitochondrial apoptotic pathway activated by UVB exposure in human keratinocytes. Also, we indicate that the antioxidant effect of SHM such as the activation of Nrf2-mediated HO-1 signaling and the inhibition of intracellular ROS generation affected to its anti-apoptotic effects in UVB-exposed human keratinocytes.

The transcription factor, NF-κB is a upstream cellular signaling pathway responsible for various functions, including stress responses, apoptosis, cell proliferation, and innate immune responses towards a variety of internal and external stimuli^38,39^. When the existing antioxidant defense mechanisms fail to regulate excess of ROS levels, the cells advance into a state of oxidative stress, which enables the activation of NF-κB pathways^10,40^. Many researchers have reported that UVB exposure can induce the oxidative stress by producing the excessive intracellular ROS generation and it causes the activation of NF-κB signaling^41,42^. So, the inhibition of NF-κB signaling is important for the UVB protective capacities as well as the antioxidant capacities. Indeed, there are many reports about the roles of various biomaterials as UVB protectors on the inhibition of NF-κB activation with their antioxidant effects^41–43^. Our results also exhibited that SHM dose-dependently inhibited the UVB exposure-induced activation of NF-κB signaling as well as the reduction of abnormal intracellular ROS generation in human keratinocytes. Moreover, the activation of Nrf2-mediated HO-1 signaling leads to inhibition of NF-κB nuclear translocation-mediated oxidative stress and apoptosis through HO-1 end-products^33,44,45^. In addition, UVB exposure causes the reduction of nuclear Nrf2 and cytoplasmic HO-1 expression levels as well as the reduction of cell viability, the increment of intracellular ROS generation, apoptosis, and NF-κB activation in keratinocytes^46,47^. With our results and the reports mentioned in the above, the role of SHM on Nrf2/HO-1 signaling might be important for its cytoprotective and anti-apoptotic effects in UVB-exposed human keratinocytes. Indeed, as revealed in Fig. 4, the cytoprotective and the antiapoptotic effects of SHM were effectively abolished by the application of ZnPP, a HO-1 inhibitor in human keratinocytes, compared to the only UVB-exposed cells. These results first suggest that the SHM-induced the activation of Nrf2/HO-1 signaling are required for its cytoprotective and anti-apoptotic effects in UVB-exposed human keratinocytes. Additionally, our results and previous studies have reported that *S. horneri* and its derives, HTT and/or Apo-9′-fucoxanthinone increased the UVB exposure-inhibited Nrf2/HO-1 activation in human keratinocytes (data not shown) and inhibited the NF-κB activation through the induction of Nrf2/HO-1 signaling in LPS-activated macrophages^16,20^. With these results, we can suggest that the Nrf2/HO-1 activation induced by SHM containing HTT and Apo-9′-fucoxanthinone is required for its UVB-protective effect in human keratinocytes.

UVB exposure causes the dysfunction of skin epidermal barrier and skin dryness^42^. UVB-exposed cells showed the decreased levels of LEKT1, involucrin and filaggrin, which are essential for the formation of the epidermal barrier, while increased KLK5, PLA-2 and PAR-2, which cellular adherence, and regulate skin shedding^29^. Normally, UVB exposure-increased KLK5 level reduces the expression of LEKT1, then activates PLA-2 that releases several lipid mediators, and subsequently results in PAR-2 activation, further activating NF-κB signaling^48^. Generally, claudins, occludin, and ZO-1 are involved in maintaining the normal functionality of the skin barrier and collectively form tight junctions in the stratum granulosum^49,50^. These skin barrier molecules such as claudin-1, claudin-4, occludin, and ZO-1 directly influence the ion permeability of keratinocytes while claudin-1 in particular is responsible for its influence on the stratum corneum proteins regulating the skin moisturization function^49^. Our results showed that UVB exposure reduced the expressions of the LEKT1, involucrin, and filaggrin, and KLK5, PLA-2, and PAR-2 expression levels in human keratinocytes (Fig. 5). Interestingly, they were modulated by the pre-treatment of SHM. Moreover, SHM effectively up-regulated the expression levels of tight junction-related molecules such as claudins, occludin, and ZO-1 reduced by UVB exposure in human keratinocytes. With the results, the current study first demonstrated a critical role of SHM with the improvement of skin dryness and barrier dysfunction in UVB-exposed human keratinocytes.

In conclusion, our data suggest that SHM protects human keratinocytes by the activation of Nrf-2/HO-1 signaling against oxidative stress and cellular damages and improved the skin barrier dysfunction and skin dryness caused by UVB exposure. Taken together, SHM may be useful as a potential candidate for developing cosmetics with UVB protective effects (Supplementary information S1).

## Supplementary Information

Supplementary Information.
