# Supplementary material for: UVB protective effects of Sargassum horneri through the regulation of Nrf2 mediated antioxidant mechanism
Source: Sci Rep. 2021 May 11;11:9963. doi: 10.1038/s41598-021-88949-3 (PMC8113259; doi:10.1038/s41598-021-88949-3)
Supplement: Supplementary file 1 — Supplementary Information. [file 41598_2021_88949_MOESM1_ESM.docx]

Supplementary materials

**UVB protective effects of *Sargassum horneri* through the regulation of Nrf2 mediated antioxidant mechanism**

Eui Jeong Han^1,2,†^, Seo-Young Kim^3,†^, Hee-Jin Han^2^, Hyun-Soo Kim^4^, Kil-Nam Kim^3^, Ilekuttige Priyan Shanura Fernando^5,6^, Disanayake Mudiyanselage Dinesh Madusanka^2^, Mawalle Kankanamge Hasitha Madhawa Dias^2^, Sun Hee Cheong^2,6^, Sang Rul Park^7^, Young Seok Han^8^, Kyounghoon Lee^9,*^, Ginnae Ahn^2,6,**^

^1^Research Center for Healthcare and Biomedical Engineering, Chonnam National University, Yeosu 59626, Republic of Korea

^2^Department of Food Technology and Nutrition, Chonnam National University, Yeosu 59626, Republic of Korea

^3^Chuncheon Center, Korea Basic Science Institute, Chuncheon, 24341, Republic of Korea

^4^National Marine Biodiversity Institute of Korea, Janghang-eup, Seocheon 33662, Republic of Korea

^5^Control Center for Aquatic Animal Diseases, Chonnam National University, Yeosu 59626, Korea

^6^Department of Marine Bio-Food Sciences, Chonnam National University, Yeosu 59626, Republic of Korea

^7^Estuarine & Coastal Ecology Laboratory, Department of Marine Life Sciences, Jeju National University, Jeju 63243, Republic of Korea

^8^Neo Environmental Business Co., Daewoo Technopark, Doyak-ro, Bucheon 14523, Republic of Korea

^9^Division of Fisheries Science, Chonnam National University, Yeosu 59626, Republic of Korea

^†^These authors equally contributed to the work.

^*,**^These authors equally contributed to the work.

^*^Co-corresponding author:

**K. Lee**, Department of Marine Technology, Chonnam National University, Yeosu 59626, Republic of Korea. Tel: +82 616597124. Fax: +82 616597219. E-mail: [khlee71@jnu.ac.kr](mailto:khlee71@jnu.ac.kr)

^**^Co-cocorresponding author:

**G. Ahn**, Department of Marine Bio-Food, Chonnam National University, Yeosu 59626, Republic of Korea. Tel: +82 616597213. Fax: +82 616597219. E-mail: gnahn@jnu.ac.kr


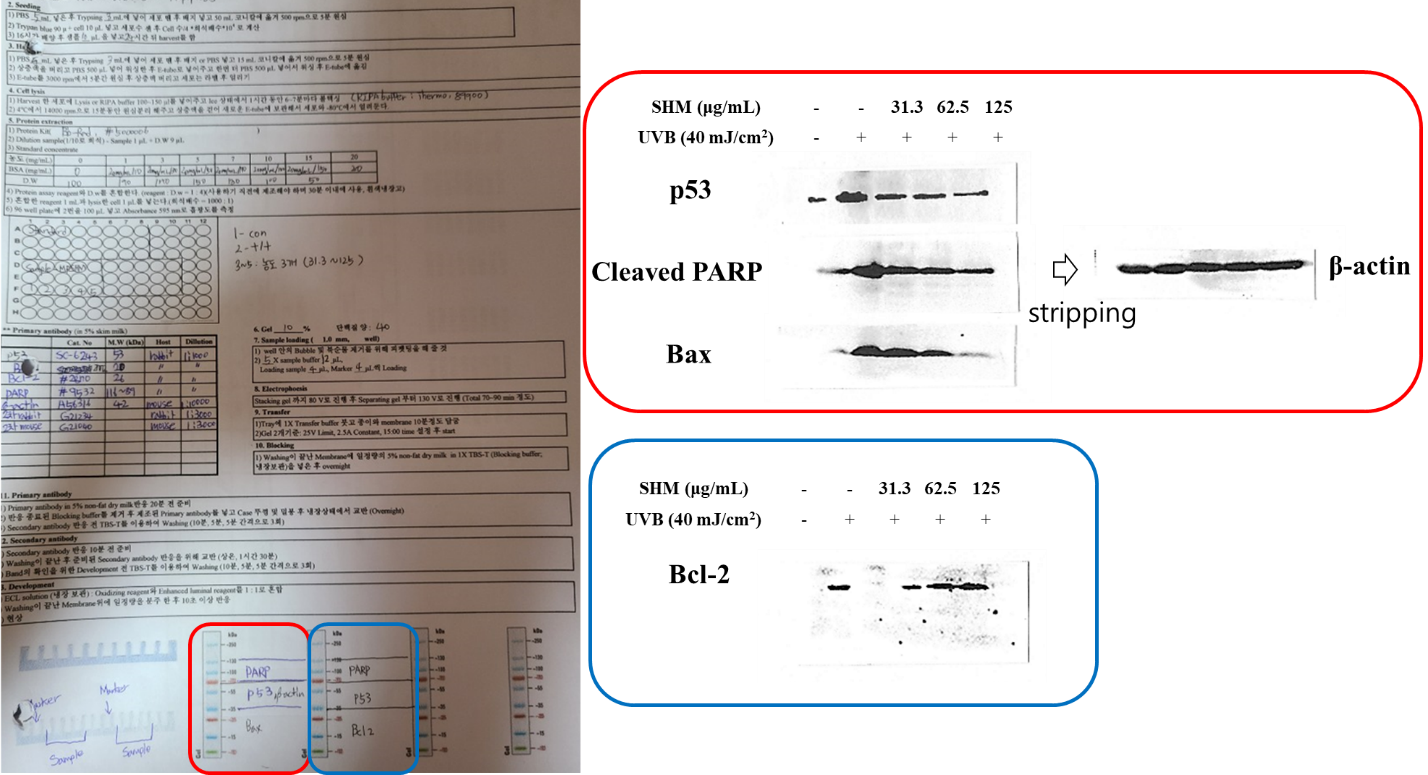


**Supplemental Figure 1.**

The original western blot result of apoptosis mediated molecules and datasheet of analysis. Before hydration with antibodieies, the western blot membrane was cut off to appropriate for the molecular weight of each antibodies.


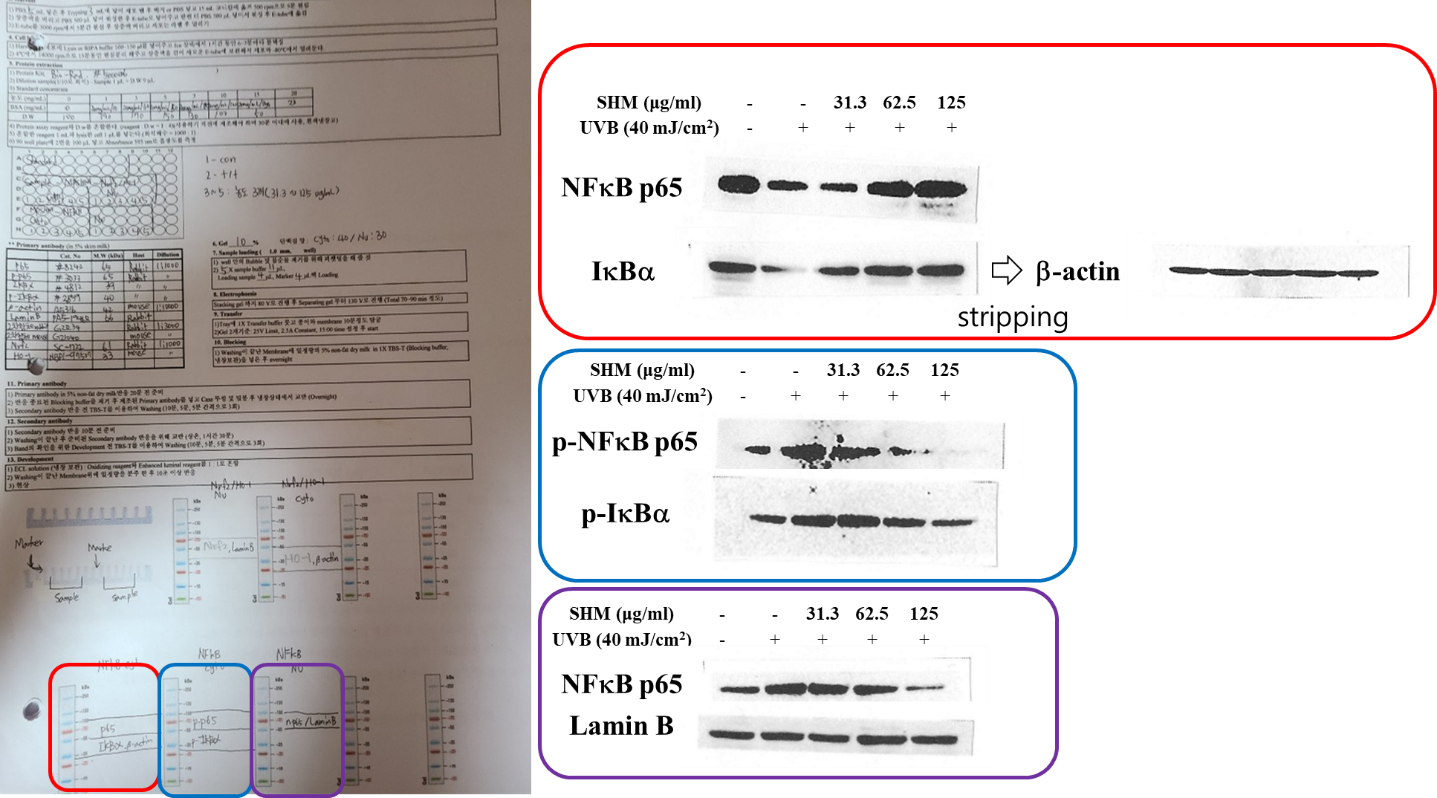


**Supplemental Figure 2.**

The original western blot result of NF-κB molecules and datasheet of analysis. Before hydration with antibodieies, the western blot membrane was cut off to appropriate for the molecular weight of each antibodies.

**
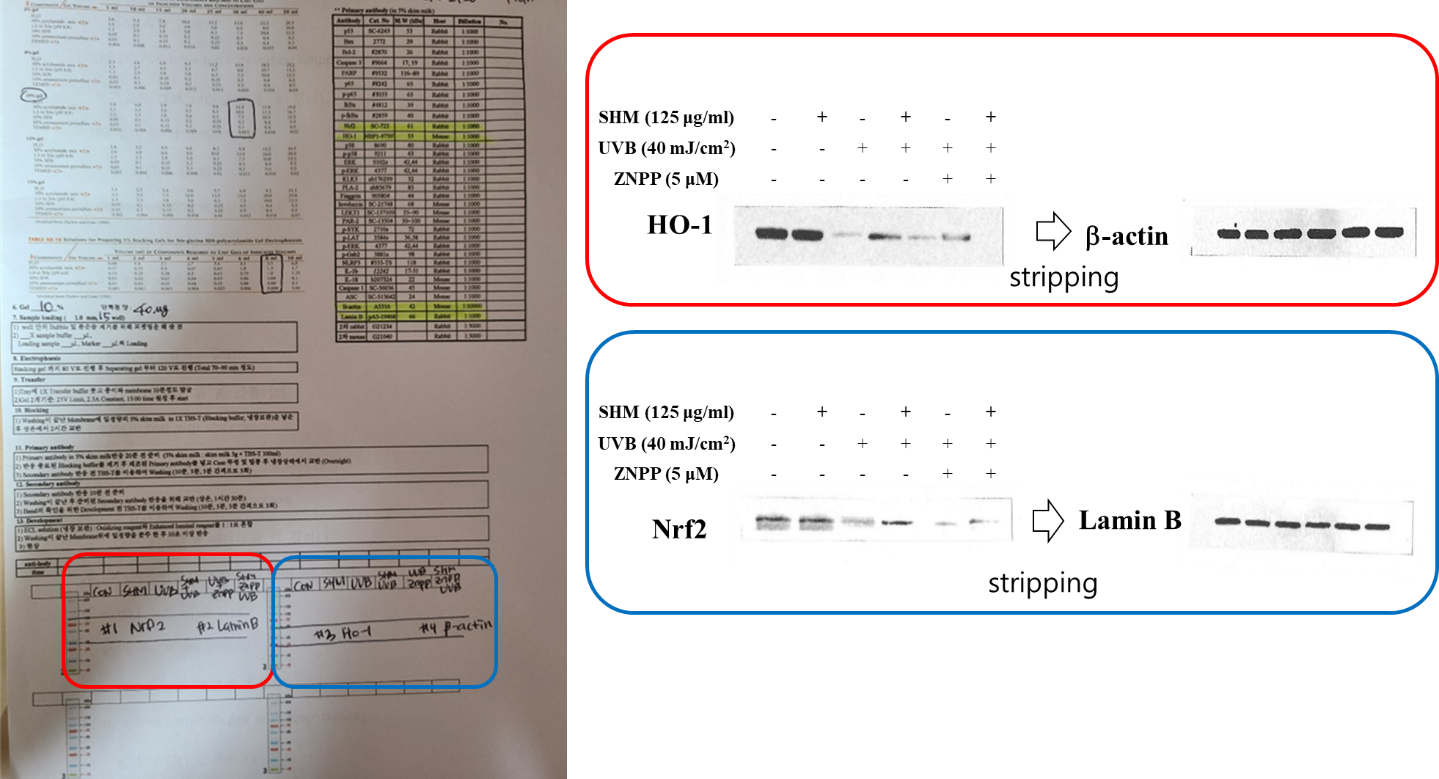
**

**Supplemental Figure 3.**

The original western blot result of Nrf2/HO-1 molecules in the presence of ZNPP, an HO-1 inhibitor, and datasheet of analysis. Before hydration with antibodieies, the western blot membrane was cut off to appropriate for the molecular weight of each antibodies.

**
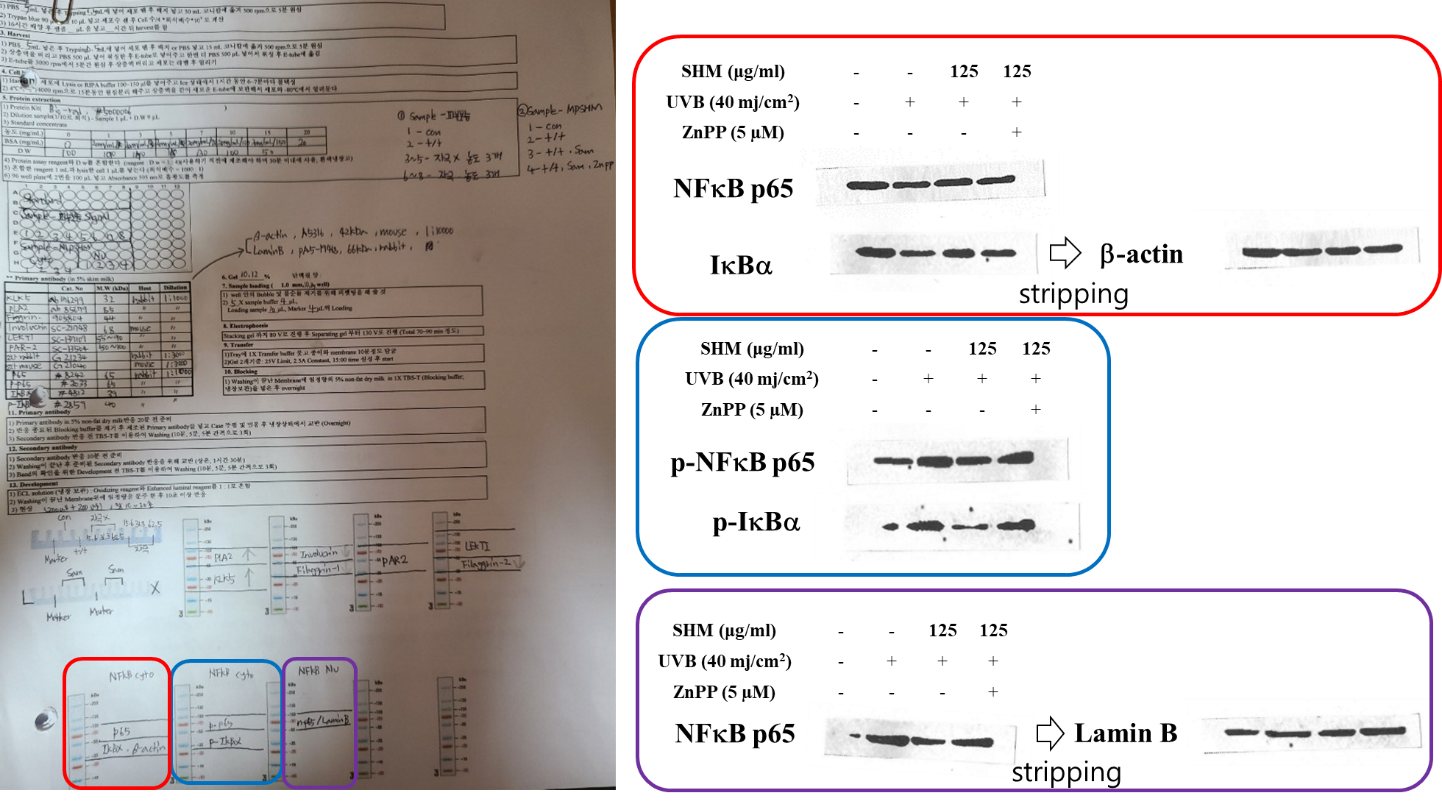
**

**Supplemental Figure 4.**

The original western blot result of NF-κB molecules in the presence of ZNPP and datasheet of analysis. Before hydration with antibodieies, the western blot membrane was cut off to appropriate for the molecular weight of each antibodies.

**
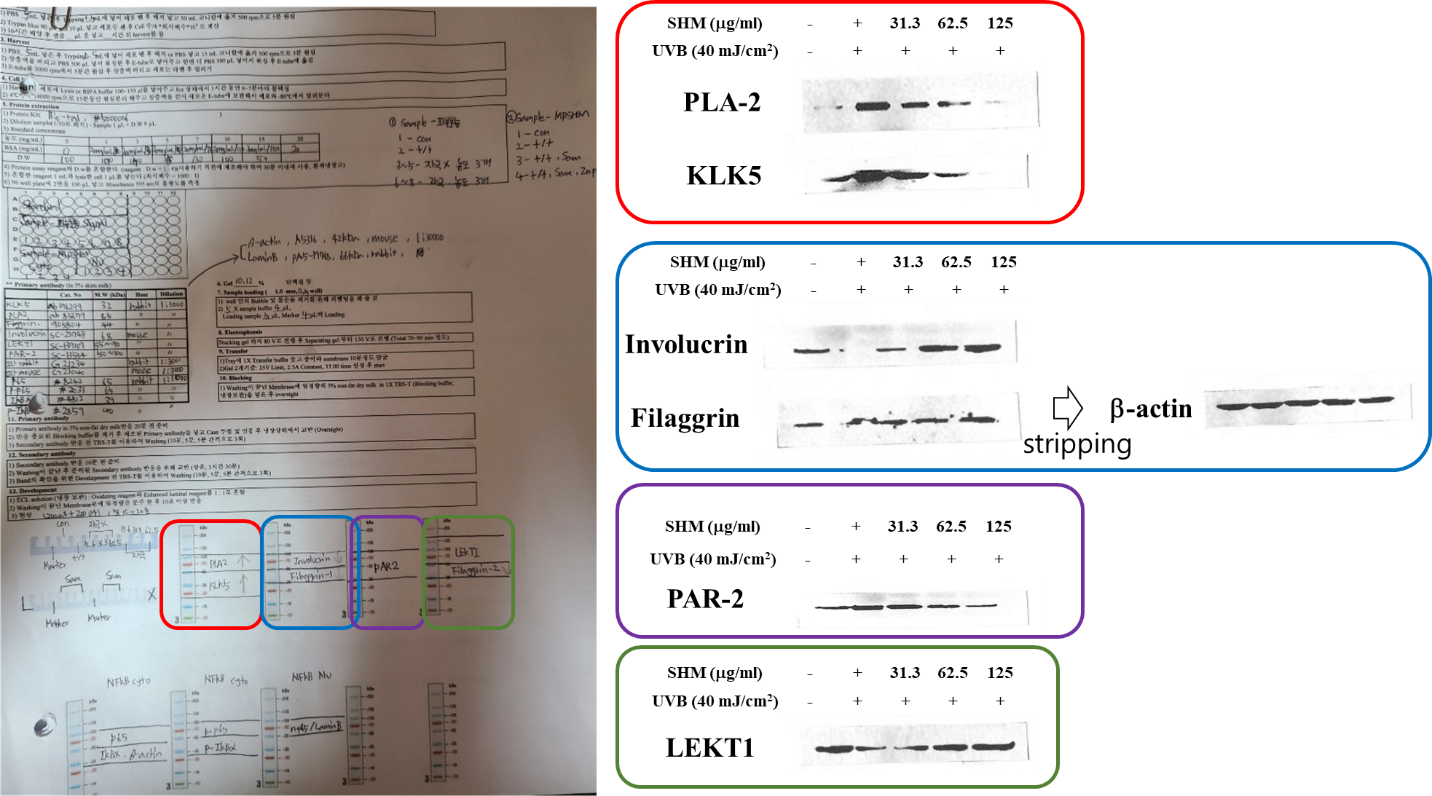
**

**Supplemental Figure 5.**

The original western blot result of skin moisture mediated molecules and analysis datasheet of analysis. Before hydration with antibodieies, the western blot membrane was cut off to appropriate for the molecular weight of each antibodies.

**
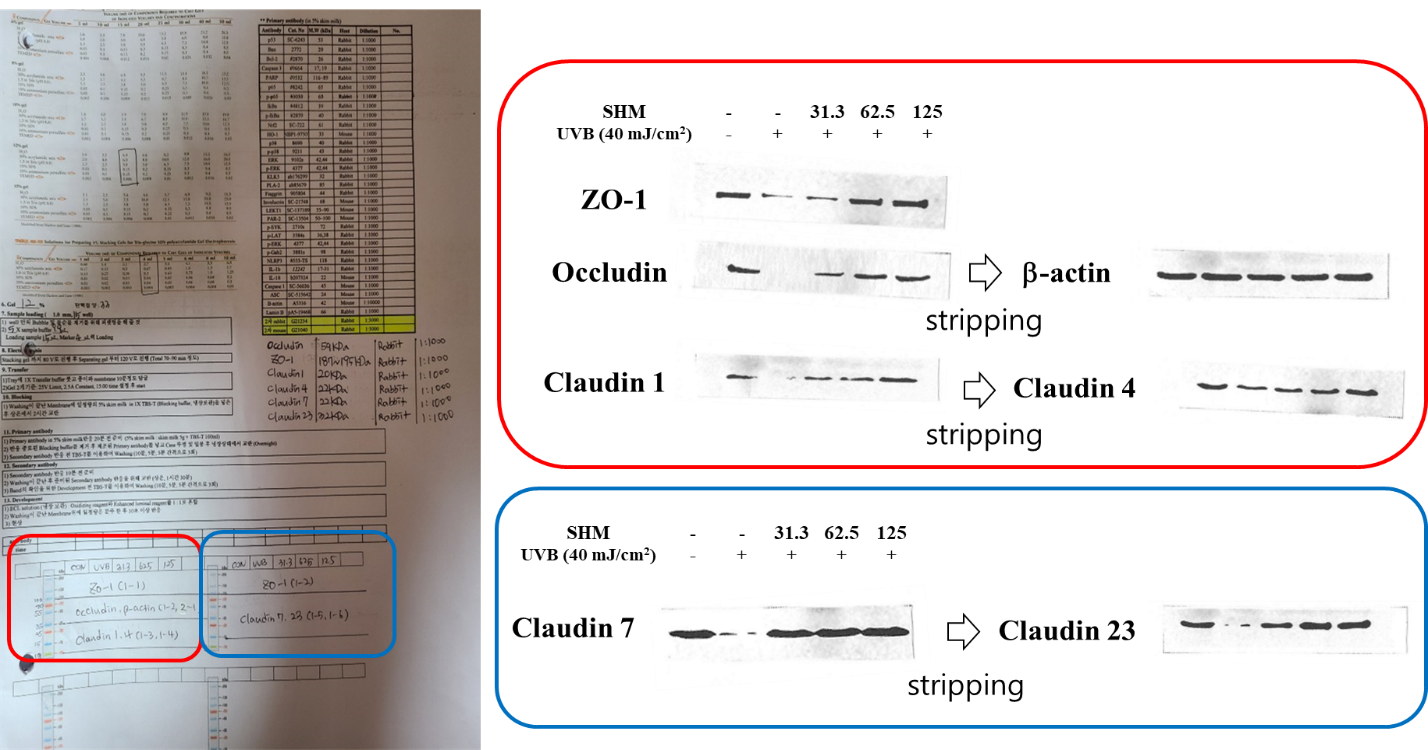
**

**Supplemental Figure 6.**

The original western blot result of skin barrier mediated molecules and analysis datasheet of analysis. Before hydration with antibodieies, the western blot membrane was cut off to appropriate for the molecular weight of each antibodies.

**
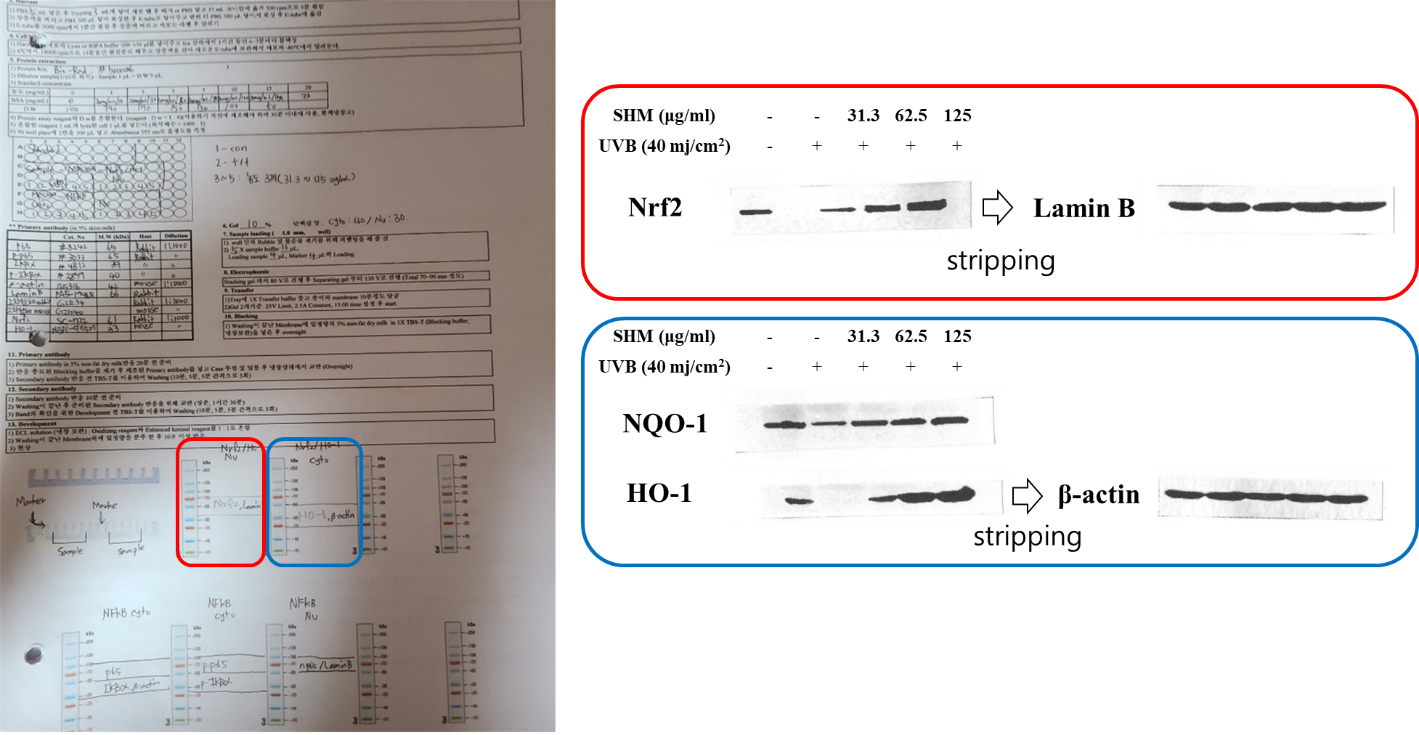
**

**Supplemental Figure 7.**

The original western blot result of Nrf2/HO-1/NQO-1 molecules and datasheet of analysis. Before hydration with antibodieies, the western blot membrane was cut off to appropriate for the molecular weight of each antibodies.
